# Paramylon, a Potent Immunomodulator from WZSL Mutant of *Euglena gracilis*

**DOI:** 10.3390/molecules24173114

**Published:** 2019-08-27

**Authors:** Laura Barsanti, Paolo Gualtieri

**Affiliations:** Istituto di Biofisica, CNR, Via Moruzzi 1, 56124 Pisa, Italy

**Keywords:** β-glucans, immune system, PAMPs, PRRs, glucan source, glucan extraction, *Euglena gracilis*

## Abstract

β-glucans, heterogeneous glucose polymers present in many organisms, have the capability to activate the innate immune system. Efficacy of activation depends on purity of the compound, molecular structure, polymerization degree, and source. One of the purest forms of crystallized β-(1–3)-glucan present in nature is the paramylon extracted from the WZSL non-chloroplastic mutant of *Euglena gracilis*, which can be processed to produce linear nanofibers capable of interacting with specific receptors present on cell membranes. The effects of these nanofibers, already investigated on plants, animals, and humans, will be analyzed.

## 1. Background

β-glucans are natural polysaccharides present in fungi, algae, bacteria, and plants, which differ in structure, size, branching frequency, and conformation. All these features influence their physiological functions and biological activity. These polysaccharides commonly consist of a main chain of β-(1,3) and/or β-(1,4)-glucopyranosyl units in non-repeating non-random order, with side chains of varying lengths [1,2,3]. β-glucans function as pathogen-associated molecular patterns (PAMPs) and can be non-specifically recognized by pattern recognition receptors (PRRs) present on the surface of the innate immune system cells [4,5]. β-glucans have a potent immunomodulating activity [6,7,8]; their action is mediated through receptors present on immune cells, such as Dectin-1 (a C-type lectin receptor), Toll-like receptors, complement receptor 3, scavenger receptor, and lactosylceramide and elicits specific biological responses [9,10,11].

Dectin-1 is indicated as the preferential receptor for linear β-(1,3)-linked glucans [12,13,14,15]. Upon linkage of the glucan effector with Dectin-1, the innate immune response is activated, which leads to production of both ROS and inflammatory cytokines, through the activation of transcription factors such as nuclear factor kappa-light-chain-enhancer of activated B cell (NF-κB), enzymes such as phospholipase C, and mitogen-activated protein kinases [10]. This ability to enhance defense mechanisms against infection and simultaneously down-regulate inflammations makes β-glucans a promising alternative to the mainstream use of immunosuppressive drugs for inflammatory diseases.

In order to avoid controversial results on the bioactivity of β-glucans, especially in clinical trials, efforts should be made to use highly purified glucans, free from components that could alter their affinity for innate immunity receptors or counteract their pro-inflammatory potency. Among linear β-(1,3)-linked glucans, paramylon extracted from the non-chloroplastic WZSL mutant of *Euglena gracilis* can be considered highly pure since it lacks any contamination from cellular components (membranes, proteins, pigments). Such purity is confirmed by optical and scanning electron microscope examination and by NMR spectrum, which indicates the presence of 100% glucose [2] and guarantees the absence of any non-specific immune-modulating effects. Paramylon, either in granular form or as linear β-(1,3)-linked glucan nanofibers, has been used to assess the beneficial potential of the polysaccharide and investigate the structure-function relationship and mechanism of action in animal and plant models.

We will analyze and discuss four experimental cases on which paramylon granules and paramylon nanofibers from the WZSL mutant have been tested. The four cases are

- Experimental Case 1: induction of stress resistance in *Artemia* sp.
- Experimental Case 2: activation of innate immune responses in human lymphocytes.
- Experimental Case 3: anti-fibrotic effect on CCl_4_-induced liver damage in mice.
- Experimental Case 4: increase of drought resistance and improvement of quality profile in horticultural species.

## 2. WZSL Mutant and Paramylon

**Figure 1:**
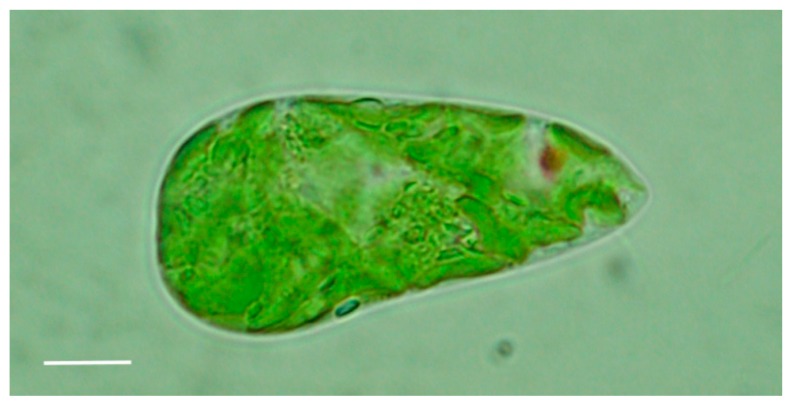
Optical microscopy image of *Euglena gracilis* wild type, showing its photosynthetic machinery. Bar: 10 μm.

**Figure 2:**
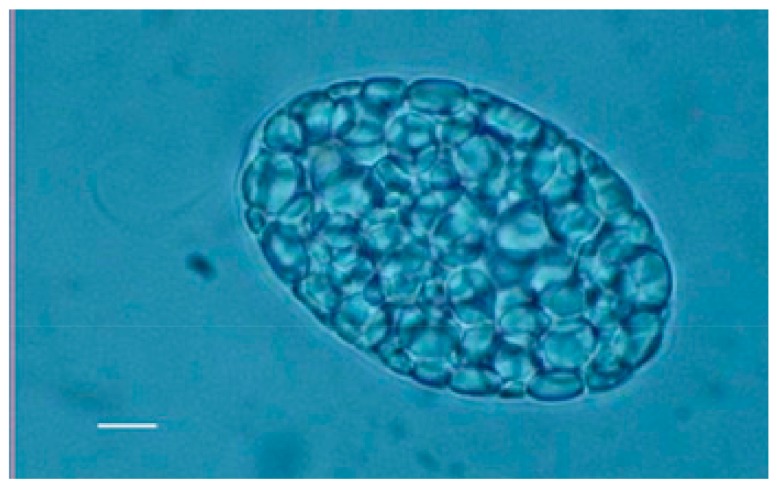
Optical microscopy image of WZSL mutant filled up with paramylon granules. Bar: 10 μm.

Both wild type (Figure 1) and WZSL mutant cells of *Euglena gracilis* (Figure 2) synthesize the storage polysaccharide paramylon, which is deposited as granules in the cytoplasm. These granules, ranging from 1 to 2 μm, have a very high degree of crystallinity, due to the concentric deposition of higher order aggregates of elemental 4–10 nm nanofibers consisting of unbranched triple helices of β-(1–3)-glucan chains. The cellular content of paramylon varies greatly with growth conditions (in the light or in the dark) and carbon source. For both cell types, under batch culture conditions and under both growth conditions, the highest concentration of the polysaccharide is reached after 24 h, with higher values in the dark respect to the light, and with glucose as best carbon source. Paramylon content of the mutant can be almost twice the content of the wild type: about 2.5 ng/cell, corresponding to 85% of the dry biomass in the mutant, respect to about 1.4 ng/cell, corresponding to 48% of the dry biomass in the wild type [16].

WZSL mutant can be cultivated aseptically using a fed-batch technique, a culture technique commonly used in fermentation industry to attain high cell densities and increase the quality of the final product, especially important for algal biomass intended for extraction of high-value pure compounds [17,18]. Fed-batch culture in the dark using Cramer and Myers medium [19], with 10g/L glucose reaches a biomass yield of 8 g L^−1^ after 20 days of cultivation, with a production of paramylon constantly higher than 80% of the dry biomass.

Thanks to their high crystallinity, paramylon granules can be extracted from the mutant cells very easily by sonication or other mechanical disruption method and purified by using a low concentration detergent to solubilize all the membrane components that could contaminate the final product [15]. Granules are recovered by low-speed centrifugation and rinsed repeatedly to remove any trace of detergent. Paramylon nanofibers are obtained by alkaline degradation of the granules, which leads to the unwinding of the ordered arrays of linear nanofibers, and to their random fragmentation in small unit recognizable by Dectin-1 receptors [12,13]. This processing improves the interaction of the β-(1,3)-linked glucan with PRRs present on cell membranes [4,5,12,13,14,15].

## 3. Experimental Cases

### 3.1. Experimental Case 1: Induction of Stress Resistance in Artemia sp.

**Figure 3:**
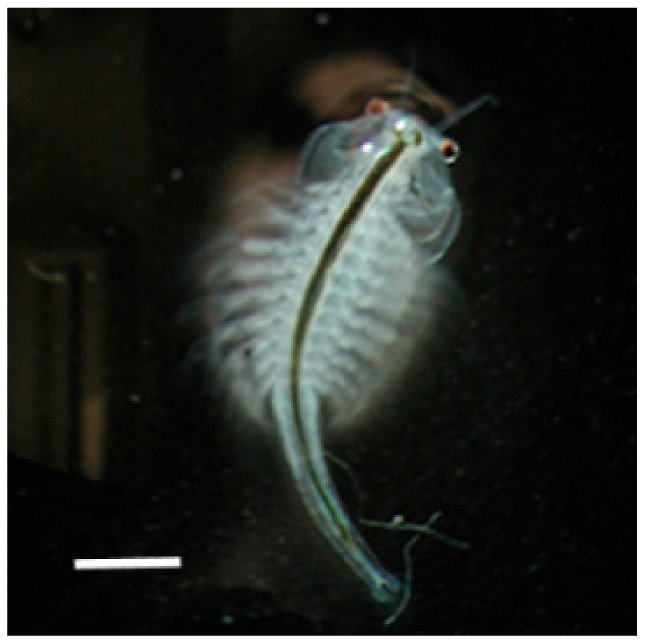
Optical microscopy image of *Artemia* sp. adult. Bar: 1 mm.

The first test of paramylon bioactivity was conducted on the brine shrimp *Artemia* sp. (Figure 3) to investigate whether this glucan could help the animals to cope with and eventually overcome stress conditions during growth [20].

**Figure 4:**
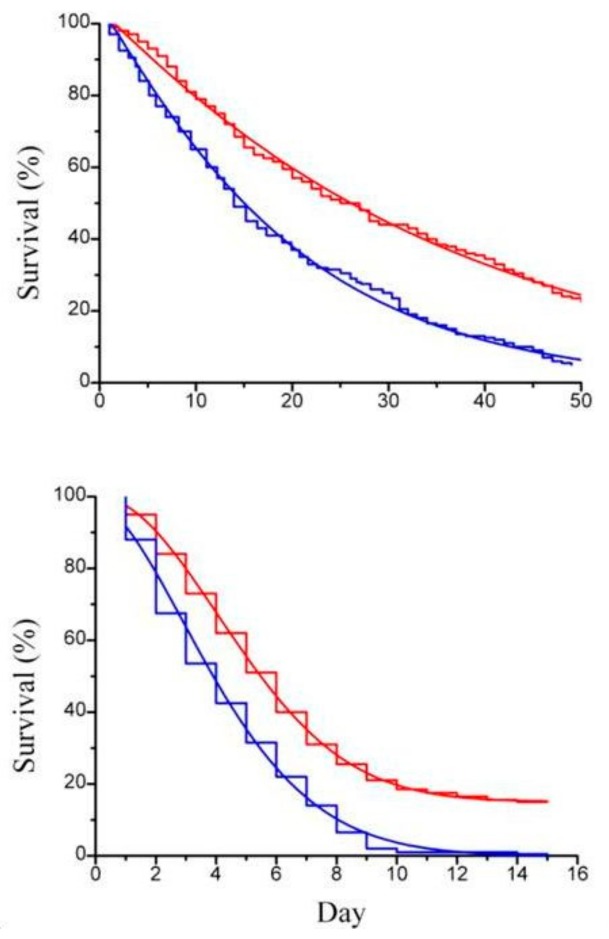
(**a**) Survival curve (step line) of *Artemia* sp.; (**b**) survival curve (step line) of *Artemia* sp. offspring with the corresponding Weibull survival functions (continuous line), with paramylon (red curve) and without paramylon (blue curve), under conditions of no daily medium replacement.

**Figure 5:**
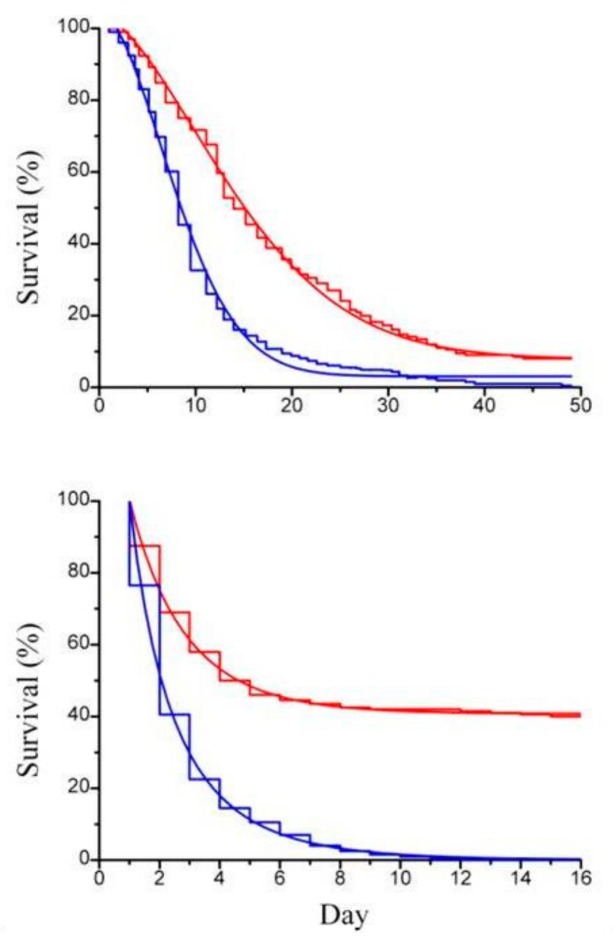
(**a**) Survival curve (step line) of *Artemia* sp.; (**b**) survival curve (step line) of *Artemia* sp. offspring with the corresponding Weibull survival functions (continuous line), with paramylon (red curve) and without paramylon (blue curve), under conditions of daily medium replacement.

Previous works by Itami et al. [21] and Lopez et al. [22] used β-glucan derived from *Bifodobacterium* sp. in the form of particulate cell wall fragment preparation as dietary addition to assess its physiological and immunological role in shrimps. In order to compare our result with the result of these authors [20,21], paramylon granules were tested suspended in the artificial sea water to rear the animals. Imposed stress condition were those connected to routine operations, such as daily medium replacement and handling, known to lower animal tolerance to diseases [23]. Paramylon added to the culture medium significantly enhanced the survival performance of both *Artemia* adults (Figure 4a) and offspring (Figure 4b) to deteriorated environmental conditions (no medium replacement) and increased the ability of the crustaceans to withstand the stress of daily medium replacement (Figure 5a,b).

Paramylon positively influenced also the number of the offspring produced, reducing the number of the encysted animals, both when medium was not replaced and with daily medium replaced, confirming its enhancing action on the reproductive success of the population.

Our results showed that paramylon treatment obtained significant higher survival percentage of the shrimps respect to the treatment with bacterial glucan; this may be due to the penetration of paramylon granules in the digestive tract of the shrimps, which enhances the production of cell activating factors in the hemocytes, increasing the phagocytic activity of the granulocytes, thus providing defense against diseases [20,21,22]. As other commercial products worldwide introduced as feed additive to improve health and performance of farmed shrimp, fish, farmed animals, and pets (e.g., MacroGard), paramylon could therefore be used as non-specific dietary additive in aquaculture, improving the nutritional value of the feed and mitigating negative effects of stressors when they occur by promoting innate immunity of the animals.

### 3.2. Experimental Case 2: Activation of Innate Immune Responses in Human Lymphocytes

**Figure 6:**
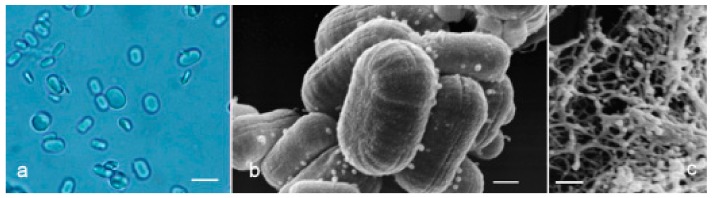
(**a**) Optical image of paramylon granules extracted from WZSL mutant. Bar: 2 μm. (**b**) SEM image of paramylon granules. Bar: 200 nm. (**c**) SEM image of paramylon sonicated and alkalized showing its linear fibrous structure. Aggregation is due to the SEM preparation procedure (gold coating thickness about 10 nm). Bar: 400 nm.

**Figure 7:**
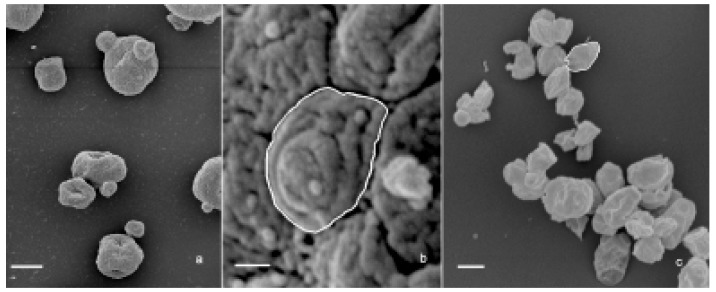
(**a**) SEM image of the aggregates present in *S. cerevisiae* preparation. Bar: 3 μm. (**b**) SEM image of an aggregate surface showing its particulate texture. The white contour defines a single particle. Bar: 200 nm. (**c**) SEM image of the irreducible particles. The white contour defines a single particle. Bar: 1 μm.

To investigate the influence of source, purity, primary chemical structure, and 3D conformation on β-glucan capacity to activate innate immune system response, we tested three different β-glucan forms i.e., paramylon granules (Figure 6a,b), paramylon nanofibers (Figure 6c), and *Saccharomyces cerevisiae*, (Figure 7a–c) processed according to Russo et al. [24], in human peripheral blood mononuclear cells (PBMC),

Evaluation of the response was carried out by measuring gene expression of inflammation-related cytokines and mediators, transactivation of relevant transcription factors, and phagocytic activity.

The three β-glucan forms greatly differ in purity, size, and 3D conformation. Paramylon granules range in size between 2–8 µm and show no contamination from other cell components (Figure 6a). Paramylon nanofibers show a degree of purity similar to that of the granules and possess a linear structure in sub-nanometric range. The preparation of *S. cerevisiae* shows aggregates of about 50 µm consisting of a patchwork of irreducible particles of heterogeneous yeast components of about 2 µm (highlighted in Figure 7b,c). According to Hunter et al. [25], these particles consist of conglomerates of several different molecules such as hexoses, proteins, lipids, and nucleic acids, which can make difficult to interpret experimental results on the effects of *S. cerevisiae* glucan.

**Figure 8:**
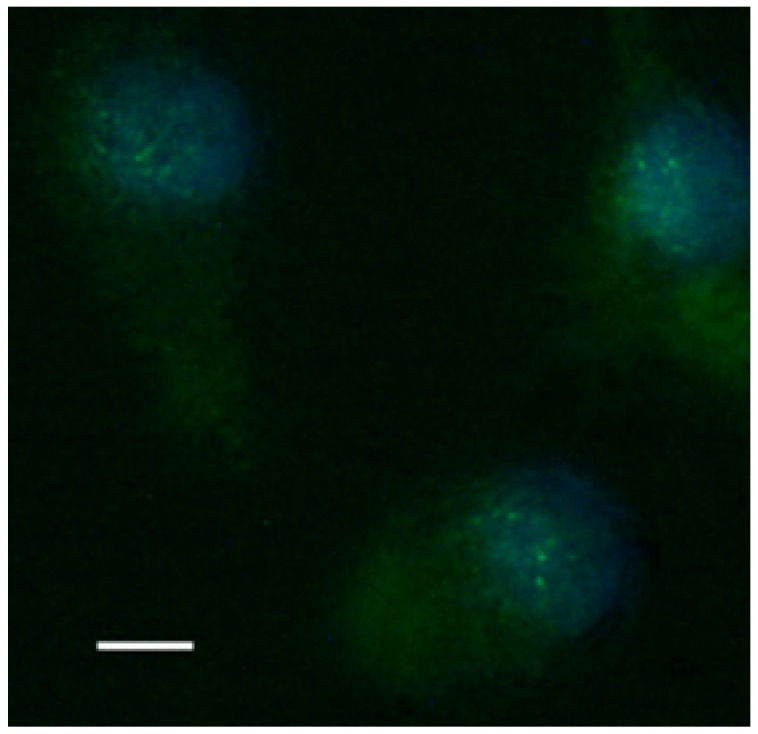
Fluorescence image of translocation of nuclear factor NF-κB in PBM cells stimulated by paramylon nanofibers. Bar 10 μm.

Paramylon nanofibers increased transactivation of NF-κB (rapid acting primary transcription factor, first responder to harmful stimuli, [26]), as shown by the evident immunofluorescence nuclear labelling of treated PBMC (Figure 8), whereas both *Saccharomyces* preparation and paramylon granules had almost no effect [24].

**Figure 9:**
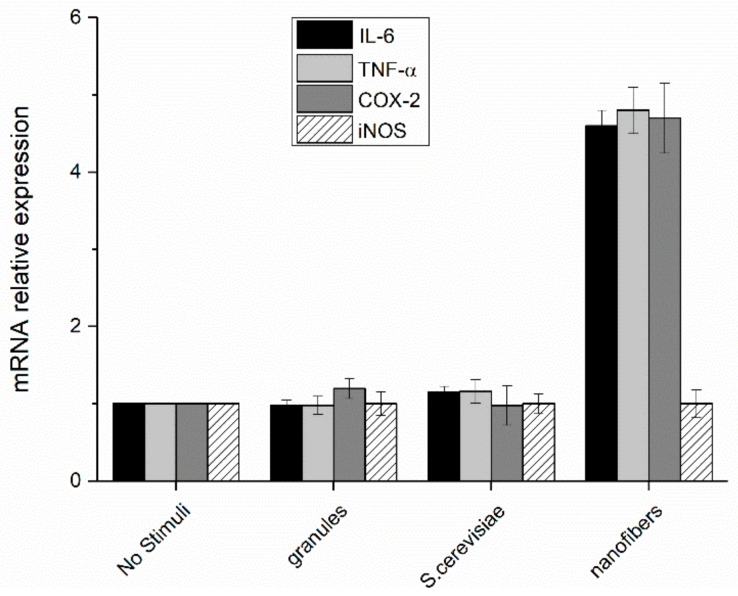
Relative expression of interleukin-6, tumor necrosis factor alpha, cyclooxygenase 2, and inducible nitric oxide synthase in PBM cells after 24 h stimulation with paramylon granules, *S. cerevisiae* preparation, and paramylon nanofibers. Results were expressed as fold-increase respect to control and plotted as the mean ± SD.

The increase in the transcription factor translocation led to a consequent increase of the expression of pro-inflammatory mediators (TNF-α, IL-6, COX-2, and iNOS) in cells treated with paramylon nanofibers, whereas the level of expression in the cells treated with paramylon granules and *S. cerevisiae* preparation did not significantly differ from that of non-treated control cells. These mediators are high inflammatory and are indispensable when priming immune responses and licensing dendritic cells (Figure 9).

**Figure 10:**
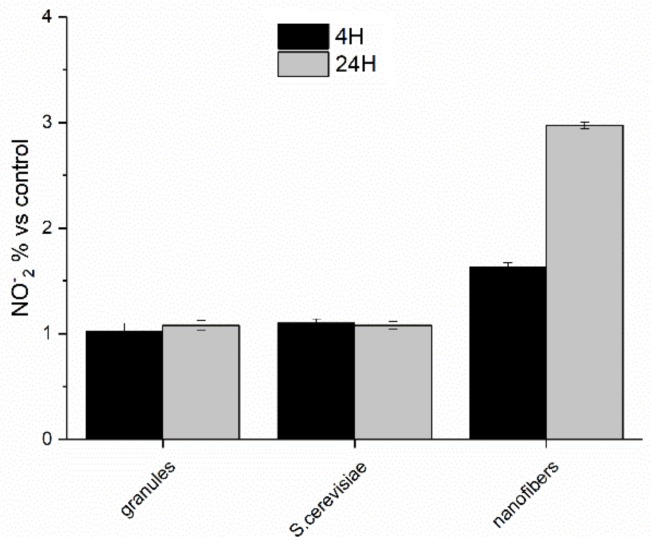
Nitric oxide production by PBMC cells stimulated with paramylon granules, *S. cerevisiae* preparation, and paramylon nanofibers 4 and 24 h, expressed as percent nitrite concentration versus control and plotted as the mean ± SD.

Paramylon nanofibers also induced the production of high level of NO (via transcription of iNOS gene), which increased from 4 to 24 h and exerted an inhibitory effect on cytokine expression via inhibition of NF-κB transactivation (Figure 10) [27], thus preventing a dangerous cytokine storm.

**Figure 11:**
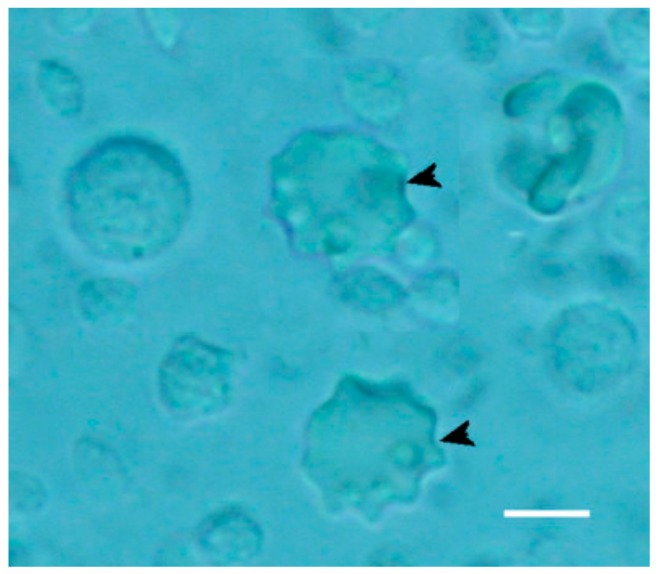
Newly differentiated dendritic cells (arrowheads) indicating activation of the innate immune system in PBM cells treated with paramylon nanofibers. Bar 10 μm.

This signaling cascade guarantees a safe activation of the innate immune system, as demonstrated by the presence of newly differentiated dendritic cells (Figure 11, arrowheads). Paramylon granules and particles of *Saccharomyces* were not absorbed to the cell surface of PBMC due to their sizes, which did not fit with Dectine-1 receptors. Though both these preparations were internalized, this phagocytic activity did not lead to any activation of the immune system, i.e., no dendritic cells differentiated [8,24].

The immune activation response obtained by paramylon nanofibers treatment fits well with evidences reported by many scientific groups [12,13,28] that the minimum β-glucan unit recognized by Dectin-1 is a 9- or 10-mer of glucose.

### 3.3. Experimental Case 3: Anti-Fibrotic Effect on CCl_4_-Induced Liver Damage in Mice

Hepatic fibrosis is one of the most severe pathology worldwide clinically associated with the eventual development of hepatocarcinogenesis and expected to become a major global burden [29]. To date, no definitive therapeutic options are available, and functional foods containing bioactive ingredients are expected to play a very important role in treating and preventing this pathology. Therefore, we explored the feasibility of paramylon nanofibers to act as a potential functional food by investigating their anti-fibrotic effect in mice with liver fibrosis induced by 4 weeks treatment of CCl_4_ administered via intraperitoneal injection [30]. Liver fibrosis induced by CCl_4_ is a reliable in vivo experimental model to investigate the progression of liver disease and monitor the effects of different therapeutic strategies [29].

One group of animals was administered 150 μL of paramylon nanofibers suspension 2 weeks before the beginning of the challenging, via voluntary suction (90 mg/kg body weight/die). The paramylon administration continued over the entire experimental period (2 + 4 weeks). Since the progression of fibrosis is mainly due to inflammation and collagen accumulation, we evaluated liver damage markers and collagen content (Picrosirius red staining and hydroxyproline determination).

**Figure 12:**
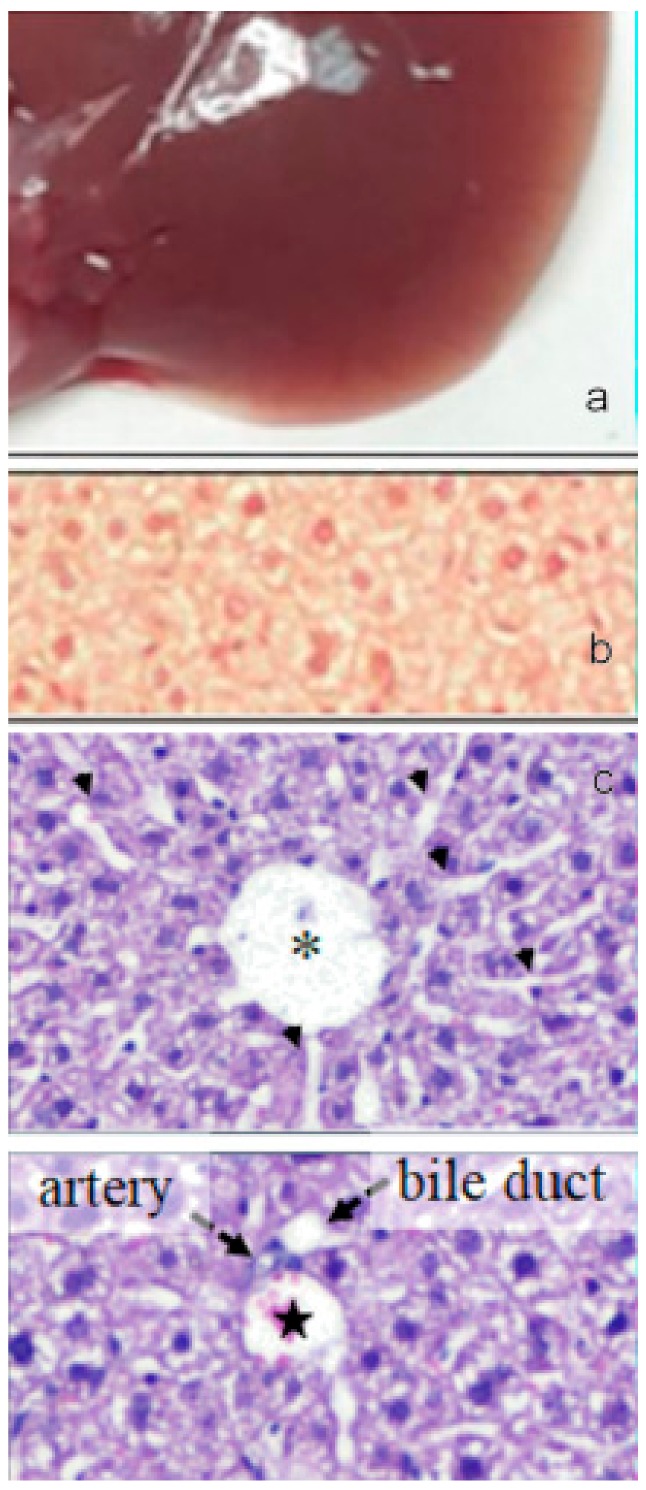
(**a**) Image of the gross morphology of the liver showing normal anatomical features in the control group. (**b**) Picrosirius red staining of liver sections: no evidence of collagen deposition in the parenchyma. (**c**) Hematoxylin-eosin staining of liver sections: normal parenchyma architecture with sinusoids (upper image) along with the portal triad (lower image), composed by a branch of the hepatic artery, a branch of the portal vein, and the bile duct. Asterisk indicates the central vein and star indicates the portal vein. Bar 50 μm.

**Figure 13:**
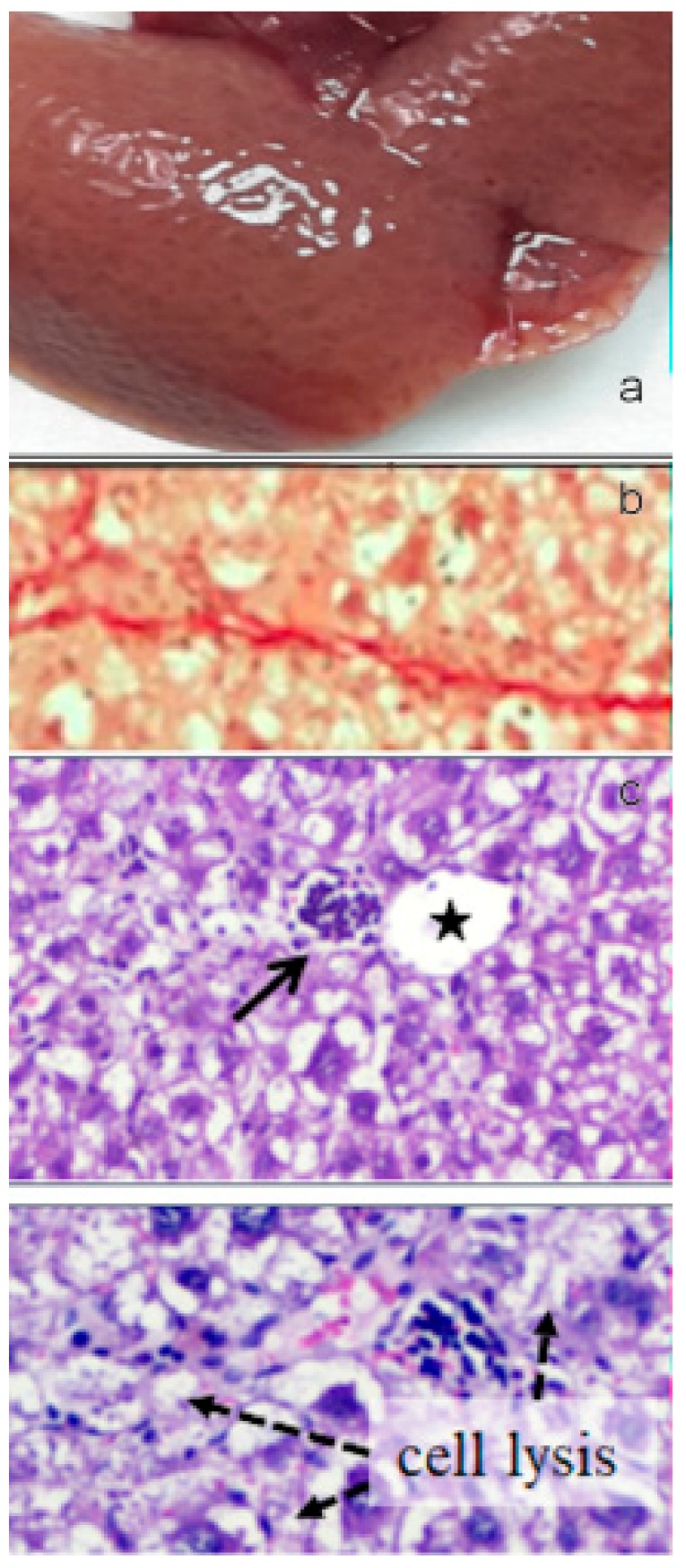
(**a**) Image of the gross morphology of the liver showing altered macroscopic characteristics in CCl_4_ challenged group with swollen and spongy appearance of the liver and lobules clearly visible on the surface. (**b**) Picrosirius red staining of liver sections: marked parenchymal deposition of collagen fibers with portal-to-portal bridging fibrosis. (**c**) Several glomerular clusters of inflammatory cells were present in the peri-portal region (upper image, arrow) and nearby focal areas of hepatocellular lysis (bottom image). Asterisk indicates the central vein and star indicates the portal vein. Bar 50 μm.

**Figure 14:**
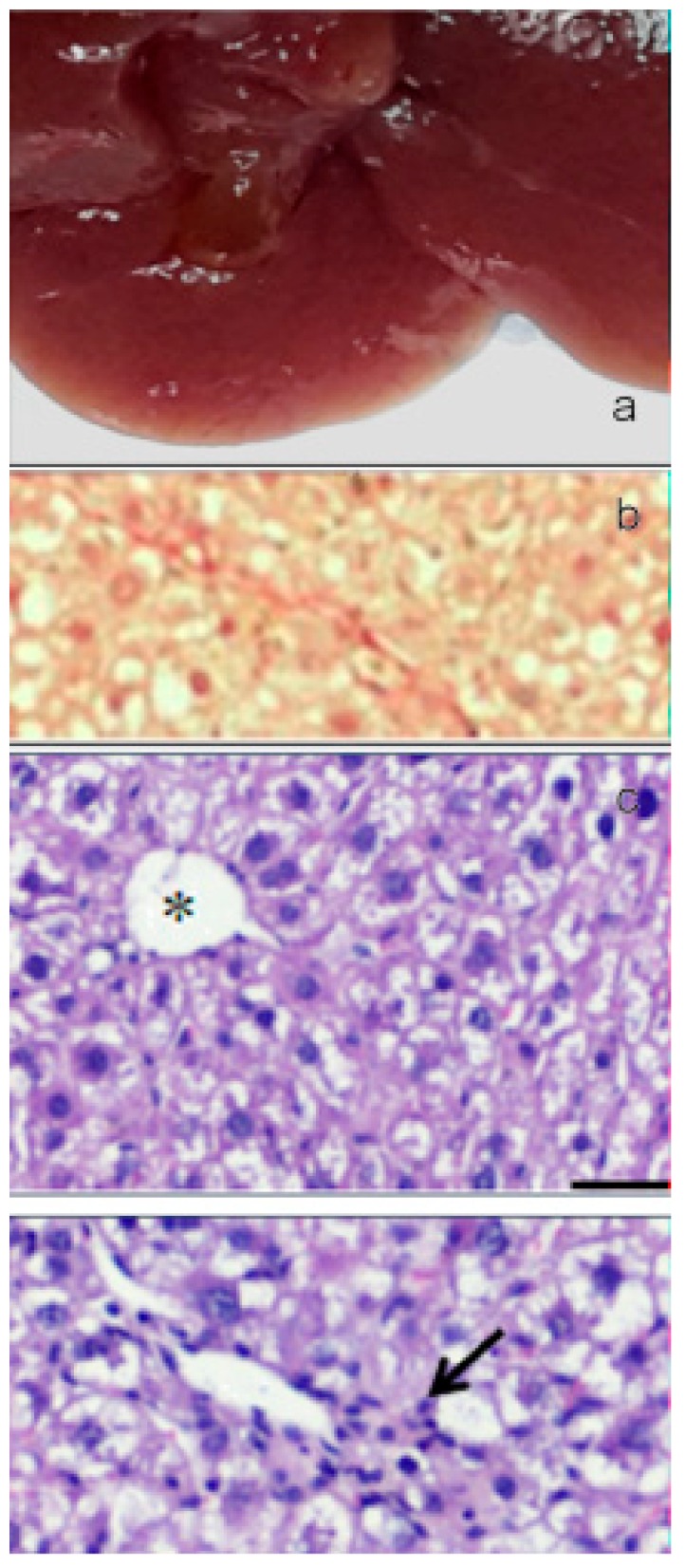
(**a**) Image of the gross morphology of the liver showing an improvement of the pathological changes induced by CCl_4_ challenge in the CCl_4_+paramylon nanofibers group in which the normal tissue consistency was almost restored. (**b**) Picrosirius red staining of liver sections: reduction of the amount of collagen deposition respect to the challenged group by paramylon nanofibers treatment. (**c**) Reversal of severe inflammation and vein congestion along with a milder vacuolization of hepatocytes (upper image). The infiltration of inflammatory cells, although still present, is greatly reduced (bottom image, arrow). Asterisk indicates the central vein and star indicates the portal vein. Bar 50 μm.

The oral administration of paramylon nanofibers ameliorated the overall clinical picture of the animals; in particular, paramylon dampened the CCl_4_-induced loss of weight and prevented the increase of aspartate aminotransferase (AST) typical of hepatocyte damage. It also reduced the hyper-echoic areas caused by the toxic treatment, restoring the normal tissue consistency and appearance. CCl_4_-treated liver had a trabecular ‘fishnet’ texture typical of mild to severe edema, while normal color and consistency was restored in paramylon treated livers, indicating a relieved hepatic injury (Figure 12a, Figure 13a and Figure 14a).

Collagen deposition in Picrosirius red staining sections of liver of paramylon treated animals was lower than in CCl_4_-treated animals (Figure 12b, Figure 13b and Figure 14b). This positive effect was confirmed by the content of hydroxyproline, which was comparable with that of the control. Moreover, hematoxylin-eosin staining of CCl_4_-damaged liver sections revealed prominent evident signs of hepatocyte suffering with diffuse distribution of inflammatory cells particularly around the portal vein. Paramylon nanofibers treatment greatly reduced the overall alteration of tissue parenchyma, and the lobular architecture was alike that non-damaged liver, with mild hepatocyte ballooning respect to CCl_4_-damaged livers and almost no necrosis regions nor infiltration of inflammatory cells (Figure 12c,d, Figure 13c,d and Figure 14c,d).

The tissue damage induced by CCl_4_ eventually leads to inflammation, which evolves as a consequence of tissue damage and is the main cause of liver injury [31]. Reduction of inflammation and collagen production by liver stellate cells is due to the inhibiting action of hepatic γδT cells upon Dectin-1 ligation by paramylon nanofibers, which promotes hepatocyte regeneration [32].

Our results cannot discriminate between a curative or preventive role of paramylon; still, they show an important protective effect of this glucan in sterile inflammation, which could be exploited for experimental therapeutics in hepatic fibrosis and inflammation. Moreover, they confirm the higher affinity of nanofibers for Dectin-1 receptor respect to granules, already tested on rat acute hepatic injury for their protective effect [33].

### 3.4. Experimental Case 4: Increase of Drought Resistance and Improvement of Quality Profile in Horticultural Species.

Drought (low water availability) is a major environmental factor limiting photosynthesis and contributing to the progressive salinization of world arable land, consequently reducing crop production all over the world [34], and tomato, one of the most economically and nutritionally important crops worldwide, is severely affected by water deficits, which compromise both fruit yield and quality. To overcome drought effects, research mainly focuses on the selection of tolerant genotypes that have less demand for water.

Paramylon nanofibers applied to the root system of tomato plants have already shown to modulate hormone levels, photosynthesis, and water use efficiency [35]. To verify the possibility of using paramylon as a novel root treatment to help tomato plants to cope with low water availability, plants of *Solanum lycopersicum* cultivar Micro-Tom, chosen because of its short life cycle (≤3 months) and small size, were challenged with drought stress with or without root treatment with paramylon nanofibers [36]. Plants were grown under controlled conditions in an aereoponics system, a soil-less air-water system particularly suited to study plant roots since roots grow suspended in a closed chamber in the dark, and the nutrient solution is directly sprayed onto them by atomizers.

**Figure 15:**
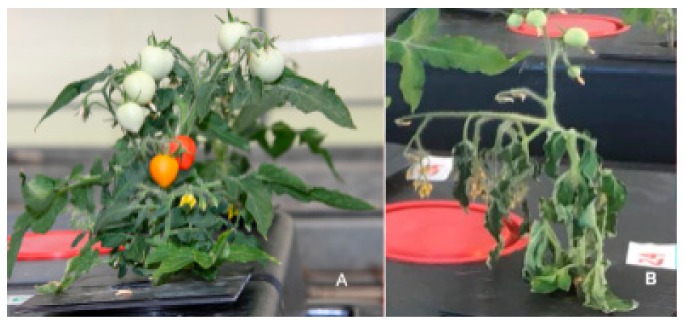
The representative features of the tomato plants (aerial parts) cultivated under water-stressed conditions coupled with root treatment with paramylon nanofibers (**A**) and under water-stressed conditions without paramylon nanofibers (**B**) 60 days after germination.

Ecophysiological, physical chemical and quality parameters were monitored throughout the life cycle of tomatoes and compared with those of well-watered, untreated plants. Flowering and fruit ripening of stressed paramylon treated plants were about 2 weeks precocious respect to untreated well-watered plants, whereas fruits of stressed untreated plants do not ripe beyond category II (Figure 15).

Thanks to paramylon action, we observed that the optimal water regimen of about 8.64 L plant^−1^ day^−1^ can be lowered down to 0.36 L plant^−1^ day^−1^. Ecophysiological parameters, (i.e., leaf water potential, stomatal conductance, and photosynthetic yield), were dramatically influenced by water stress, all of them undergoing a continuous decrease to saturation. Root treatment with paramylon nanofibers allowed all the parameters to recover to the values of well-watered plants. These results indicate an effective action of paramylon nanofibers on stomatal behavior, whose control improves water use efficiency, hence prevents dehydration. This action is associated with a transient modification of the content of main plant hormones, i.e., abscissic acid, jasmonic acid and salicylic acid [35].

**Figure 16:**
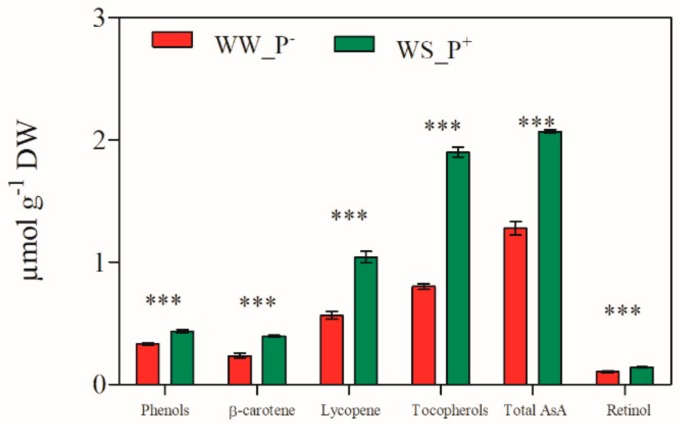
Antioxidant compounds in red ripe fruits of *S. lycopersicum* cv. Micro-Tom grown under well-watered conditions (WW_P^−^) and water-stressed conditions coupled with root treatment with paramylon nanofibers (WS_P^+^). Data are shown as mean ± SD. The significant differences are for: *** *P* < 0.001.

The great increase of physical-chemical and quality parameters such as the antioxidant compounds (Vitamin A/C/E, lycopene, β-carotene, and phenols) [35,36,37,38,39] (Figure 16), together with the increase of carbohydrates (glucose, fructose, and sucrose), in fruits of paramylon treated plants improves their nutritional value and sensory quality [40,41].

Moreover, the higher dry matter content (i.e., lower moisture) allows a better post-harvest storage capability extending the commercial period and increasing the commercial product value. These results confirm the involvement of paramylon nanofibers as elicitors of plant response to abiotic stress and their bio-stimulant activity in increasing plant adaptation capacity to environmental stimuli.

## 4. Conclusions and Outlook

Both paramylon granules and nanofibers obtained by processing the granules have been tested in our research, and nanofibers have shown the capability to effectively interact with PRRs eliciting responses in animal and plant models. Our results indicate that even though the dimensions of paramylon granules and *S. cerevisiae* particles are in the range of pathogenic bacteria (2–3 µm); still, they are not capable to interact with the Dectin-1 receptors present on the surface of immune system cells. Phagocytosis of these two preparations does not lead to any activations of the immune system since no dendritic cells differentiate [8,24]. Besides playing this important role in immune-system activation, paramylon nanofibers show also a protective effect in sterile liver inflammation, promoting hepatocyte regeneration upon Dectin-1 ligation. Moreover, they have the capability to enhance the plant defense mechanisms against abiotic stress, such as drought, by modulating hormone levels and CO_2_ diffusion from air, which eventually leads to an improvement of water use efficiency. Activation is induced only by paramylon nanofibers because they consist of a moiety of 9- or 10-mer of glucose, the minimum β-glucan recognized by Dectin-1 receptor [12,13,28].

The WZSL *Euglena* mutant can be considered an effective and desirable producer of paramylon due to its ability to convert and conserve glucose (the best carbon source for WZSL mutant, [16]) in the paramylon granules at a faster rate than the wild type, and with about half of the glucose added to the culture converted in paramylon. It is easy to grow under controlled conditions, and the cultivation in fed-batch system with glucose addition allows the production of a very high amount of paramylon per gram of dry biomass, i.e., 80%. All these features together with the experimental evidences of paramylon effects provide a reliable framework to further investigate the structure-function relation of β-(1,3)-linked glucans, offering the possibility to test a high purity and well-established preparation with no unexpected or negative side effects on different models, and opening new routes of protection and treatment against diseases and stress agents.
